# Glucose-Responsive Gene Delivery at Physiological pH through Tertiary-Amine Stabilized Boronate-PVA Particles Synthesized by One-Pot Reaction

**DOI:** 10.3390/pharmaceutics13010062

**Published:** 2021-01-06

**Authors:** Mangesh Morey, Akshay Srivastava, Abhay Pandit

**Affiliations:** CÚRAM, SFI Research Centre for Medical Devices, National University of Ireland, H91 W2TY Galway, Ireland; mangeshbiotech@gmail.com (M.M.); axay80@gmail.com (A.S.)

**Keywords:** glucose-responsive gene delivery, boronic acid, free radical polymerization, drug delivery

## Abstract

We report a physiologically stable and cytocompatible glucose-responsive nonviral gene delivery system made up of boronate functionalized polymeric material. Herein, we utilize boronate *cis*-diol interactions to develop a glucose-responsive submicron particle (SMP) system. The stability of the boronate interaction at a physiological pH was achieved by copolymerization of dimethyl aminoethyl methacrylate (DMAEMA) with acrylamidophenylboronic acid (AAPBA) and the formation of a complex with polyvinylalcohol (PVA) which is governed by *cis*-diol interactions. The shift in hydrodynamic diameter of SMPs was observed and correlated with increasing glucose concentrations at a physiological pH. Optimal transfection was observed for a 5 µg dose of the gaussia luciferase reporter gene in NIH3T3 cells without any adverse effect on cellular viability. The destabilization of the AAPBA–PVA complex by interacting with glucose allowed the release of encapsulated bovine serum albumin (BSA) in a glucose-responsive manner. In total, 95% of BSA was released from SMPs at a 50 mM glucose concentration after 72 h. A two-fold increase in transfection was observed in 50 mM glucose compared to that of 10 mM glucose.

## 1. Introduction

Nonviral gene therapy is a promising therapeutic choice for many diseases [1,2]. However, low transfection is one of the main barriers associated with nonviral gene carriers [1,2,3]. This challenge can be solved by the use of a stimulus-responsive delivery system [4]. Glucose-responsive drug delivery systems represent up-and-coming applications in diabetes therapy and have attracted much more interest in recent years [5,6]. Glucose-responsive delivery systems are widely preferred stimulus-responsive delivery systems based on Glucose oxidase enzyme (GOx), glucose binding proteins (GBPs), and phenylboronic acid (PBA) [6]. The use of these glucose oxidase-based systems is minimal, as enzyme activity can be reduced over time [7]. Concanavalin A is a natural GBP class that is one of the most widely used lectins for glucose-responsive insulin delivery. The problem with concanavalin-based systems is that they are inflammatory [8]. PBA is a synthetic molecule that reversibly binds to 1,2- or 1,3-*cis*-diols, including many kinds of sugars, to form cyclic esters.

**Figure 1:**
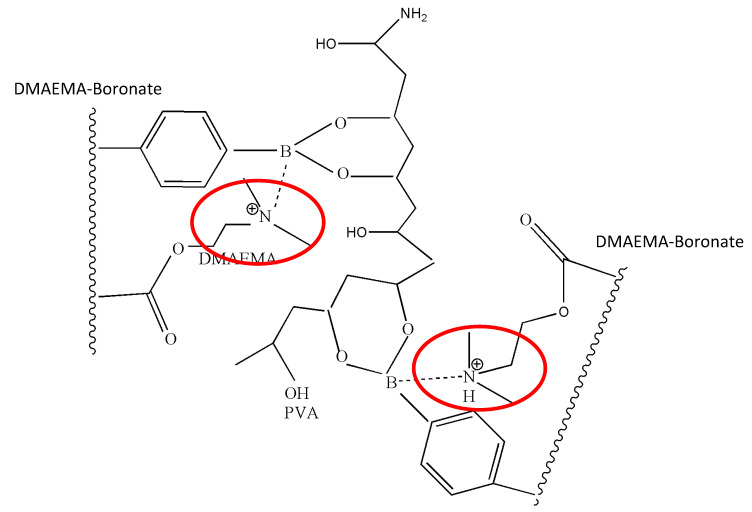
Schematic representation of stabilization of boronate by tertiary amine of dimethyl aminoethyl methacrylate (DMAEMA) in a submicron particle (SMP) system. The pKa of boronate was reduced by the addition of amino group in the vicinity of boronic acid by the formation of a coordination bond between nitrogen and boron.

Recently, boronate-based systems have been reported for their potential applications as saccharide [9], optical [10], and conductivity sensors [11,12], and also in drug release systems [13,14,15]. Boronate-based systems have been widely preferred because of their stability, efficiency, and ease of use. In aqueous media, phenylboronic acid forms a pH-dependent equilibrium between the nonionic trigonal boronic acid and anionic tetrahedral boronate. Boronate provides a reversible interaction with *cis*-diol-containing carbohydrates, nucleotides, nucleoside, and glycoproteins [16]. It has been used to develop glucose-responsive delivery systems for closed-loop insulin delivery [6]. However, boronate-based glucose-responsive polymers have been reported to remain functional only in alkaline media and not at physiological pH [17]. This is due to the high pKa value of the boronate moiety. Hence, boronate’s optimal use in biomedical engineering is restricted due to the limitations of boronate groups’ inactivity at physiological pH (7.4) [17,18,19,20]. Several methods have been developed to reduce the pKa of the boronate group to enhance its functional sensitivity at physiological pH. One such approach involves introducing an amino group in the vicinity of boronic acid to decrease the boronate group’s pKa value by forming a coordination bond between nitrogen and boron (Figure 1). The effect could be increased by introducing a tertiary amine into the boronate system [21]. Boronate-containing polymeric systems also showed adverse effects on cell metabolic activity [22]. These problems still need to be resolved before their potentially significant contribution to clinical applications can be recognized.

The goal of the study is to develop a boronate-based glucose sensitive gene delivery system. As per our knowledge, this is the first of its kind research to report the glucose-responsive gene delivery system. Additionally, we have made boronate active at physiological pH and made this synthesis very simple by using a single-pot reaction. To achieve this, we hypothesized that copolymerization of the boronate-containing monomer with dimethyl aminoethyl methacrylate (DMAEMA) and the complexation of a boronate moiety with polyvinyl alcohol (PVA) in a single reaction mixture could provide an efficient method for the fabrication of boronate-containing particles for delivery in physiological conditions. Herein, we fabricate boronate-PVA submicron particles (SMPs) and characterize the release of bovine serum albumin (BSA) as a standard biomolecule at different glucose concentrations. The SMPs were validated for glucose-responsive delivery of gaussia luciferase (GLuc) polyplexes which transfected an NIH3T3 fibroblast cell line without adversely affecting cellular viability.

## 2. Materials and Methods

### 2.1. Synthesis of 3-Acrylamidophenyl Boronic Acid (AAPBA)

**Scheme 1:**
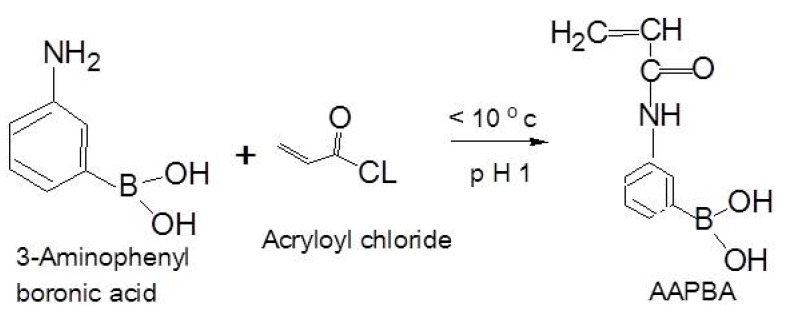
Synthesis of acrylamidophenylboronic acid (AAPBA)**.**

AAPBA was synthesized from 3-aminophenylboronic acid, as previously reported [23]. The synthesis of AAPBA was carried out, as expressed by the following reaction (Scheme 1).

Briefly, 3-aminophenylboronic acid (1.72 gm, 10 mM) was dissolved in 20 mL NaOH and cooled in an ice bath. Acryloyl chloride (1.6 mL) was then added, and the reaction was stirred for one hour. Following this, the HCl (2M) was then added to adjust the solution to pH 1, which caused the precipitation of the product in the form of a white powder. The product was filtered on the sintered glass funnel and washed with 50 mL cold distilled water. The precipitated product was collected and dissolved in the water heated at 60 °C and, finally, the dissolved precipitate was kept at 4 °C overnight. The monomer crystals were formed and washed with cold water several times and finally dried under vacuum.

### 2.2. Synthesis of AAPBA-PVA SMPs by Oil Emulsion Method

AAPBA-PVA SMPs were synthesized by the oil emulsion method by mixing two solutions. Solution A was prepared by mixing modified boronate monomer AAPBA (20 mg, 25 mM), DMAEMA (20 µl, 30 mM), 2-hydroxyethyl methacrylate (HEMA), (150 µl, 300 mM), and 10 µl *N*,*N*,*N*’,*N*’-Tetramethylethane-1,2-diamine (TEMED) was added to this solution just before the initiation of polymerization. Solution B was prepared by adding PVA (20 mg, 50 µM) and 10 mg ammonium persulfate (APS). The homogenous oil phase was prepared by adding 3.8 mL of paraffin oil and 50 µl of emulsifier Tween 20 and 150 µl of Spam 80. Free radical polymerization was initiated by mixing solutions A and B, which were immediately added dropwise in the oil phase and incubated overnight at room temperature. The emulsion was then centrifuged at 11,000 rpm for 15 min, and the pellet was washed twice with *n*-hexane followed by distilled water. The final product was freeze-dried and stored at 4 °C.

### 2.3. Physiochemical Characterization

Synthesis of AAPBA was further characterized by proton nuclear magnetic resonance (^1^H NMR) and boron nuclear magnetic resonance (^11^B NMR) by an Agilent-NMR5-vnmrs500 instrument (Agilent Technologies Ireland Limited, Cork, Ireland) using DMSO as a solvent. The synthesis of both AAPBA and SMPs was confirmed by Fourier-transform infrared spectroscopy (FTIR) analysis (FTIR—Varian 660-IR, Agilent Technologies Ireland Limited, Cork, Ireland). Freeze-dried powder of samples was used for this experiment. Scanning electron microscopy (SEM) was used for surface morphological analysis of SMPs. The gold coated samples were visualized under SEM (Hitachi S-4700 Scanning Electron Microscope, Daresbury, Warrington WA4 4AB, UK). Surface charge on the SMPs was determined by zeta sizer (Malvern, Nano-ZS90, Malvern, UK).

### 2.4. Functional Integrity of Boronate

Curcumin forms a red complex with boronate and color formation can be observed by the thin layer chromatography (TLC) method [24]. To prepare the curcumin stain, 100 mg of curcumin was dissolved in a 100 mL solution of ethanol with 2 N HCl (99:1 *v*/*v*). Boronic acid was dissolved in methanol and a single spot of each sample was placed using a glass “spotter” onto a silica TLC plate (Sigma Aldrich, City Dublin, Ireland). The TLC plate was then inserted into the curcumin stain for five seconds and then dried using a heat gun for five seconds.

### 2.5. Transfection

A range of different plasmid:polymer ratios, i.e., 1:2, 1:3.5, 1:7, 1:20, have been investigated to obtain an optimal complexation concentration of polymer for transfection. Agarose gel electrophoresis was run to determine the optimal concentration for complete complexation. The transfection experiment was carried out using NIH3T3 cells to confirm the results further. The widely used transfection reagent polyethylenimine (PEI) was used as positive control while naked plasmid was used as the negative control. Transfection of cells was analyzed after 48 h by luciferase assays using a BioLux^®^ luciferase assay kit (Biolab, Dublin, Ireland).

### 2.6. Polyplex Dose

Optimization of GLuc polyplex concentration for higher transfection was analyzed using NIH3T3 cells. In a 12-well plate, 25,000 cells/well were seeded and incubated overnight for the attachment. A few different polyplex concentrations, i.e., 0, 1, 2, 5, 25 μg were added to the cells and incubated for four hours. After four hours, the polyplex solution was replaced with regular growth medium, and the transfected cells were incubated for 48 h. After incubation, transfection was determined by luciferase assay as outlined above.

### 2.7. Glucose-Responsive Behavior

The glucose-responsiveness of the fabricated boronate SMPs was investigated by using the following techniques. Swelling of the SMPs in the presence of different glucose concentrations was analyzed with zeta sizer (Malvern, Nano-ZS90, Malvern, UK), whereas glucose-responsive release of BSA was estimated by a spectrophotometer. Furthermore, glucose-responsive gene delivery was confirmed by luciferase assay using a gaussia luciferase assay kit.

#### 2.7.1. Hydrodynamic Diameter Change by DLS Measurement 

Changes in the hydrodynamic diameter of SMPs in the presence of glucose were determined by zeta sizer (Malvern, Nano-ZS90). The SMPs (2 mg) were incubated with 5, 10, 20, and 50 mM of glucose in PBS for 30 min and the change in hydrodynamic diameter was investigated. The same amount of SMPs was also incubated with PBS only as the negative control, and the hydrodynamic diameter was calculated. All the samples were run in triplicate.

#### 2.7.2. Glucose-Responsive Release of Encapsulated BSA

To assess the glucose-responsive delivery from SMPs, BSA was encapsulated within the SMPs during their fabrication. Release of BSA in the presence of 10, 20 and 50 mM glucose concentrations in PBS over 168 h was determined. The released BSA was quantified using the Bradford assay, and the absorbance reading was measured using a varioskanflash plate reader (Thermo Scientific, Dublin, Ireland).

#### 2.7.3. Glucose-responsive Transfection

In a 12-well plate, 25,000 NIH3T3 cells/well were seeded and incubated overnight for attachment. Glucose-responsive SMPs loaded with GLuc polyplexes were incubated with the cells in a range of glucose concentrations—i.e., 0, 10, 20 and 50 mM for 24 h. The expression of the excreted luciferase protein 48 h post-transfection was quantified using the luciferase assay using a gaussia luciferase assay kit.

### 2.8. Cytotoxicity

#### 2.8.1. Metabolic Activity

The influence of the SMPs on the metabolic activity of NIH3T3 fibroblasts was quantified using the alamarBlue^TM^ cell metabolic activity assay. NIH3T3 cells were grown in a 96-well plate and treated with 10, 20, 50, and 100 µg of SMPs per well and incubated for 48 h. After 48 h, 10% alamarBlue^TM^ solution in the media was added to cells, and the plates were incubated for a further four hours at 37 °C. The supernatant’s fluorescence was measured by using a varioskan flash microplate reader (Thermo Fisher Scientific, MA, USA). The used excitation and emission wavelengths were 530–560 nm and 590 nm, respectively. The following formula was used to determine the percentage of viable cells—fluorescence of treated cells/fluorescence of untreated cells × 100.

#### 2.8.2. Live Dead Assay

In an eight-chamber slide, 10,000 NIH3T3 cells/chamber were seeded and grown to 70% confluency. AAPBA-PVA SMPs (100 μg) were added to each of the chambers for 48 h. Then, cells were washed with PBS and incubated with 10 mM calcein-AM green (Life Technologies, Dublin, Ireland) and 1 mM ethidium homodimer-1 (Life Technologies, Ireland) for 30 min. The samples were imaged by using a fluorescence microscope (Olympus BX51, Dublin, Ireland).

## 3. Results and Discussion

### 3.1. Chemical and Morphological Characterization

One-pot synthesis of AAPBA–PVA complex with DMAEMA stabilization was performed in this study. In the first step, 3-aminophenylboronic acid was modified to AAPBA through reaction with acryloyl chloride. This modification of the monomer was necessary to add an allyl moiety for free radical polymerization. The synthesis of AAPBA was confirmed by FTIR, ^1^H NMR and ^11^B NMR (Figure S1). FTIR analysis showed that the characteristic peaks at 1666 and 1636 cm^−1^ are attributable to C=O and C=C bond stretching vibrations, respectively. A typical amide band appears in the spectrum of AAPBA at 1557 cm^−1^. The absorption band at 1433 cm^−1^ is ascribed to benzene stretching vibrations (Figure S1C). The characteristic absorption of boronate took place at 1356 cm^−1^ (21). The synthesis of AAPBA was confirmed by ^1^HNMR (400 MHz, DMSO-*d*6) δ = 5.75 (1H, CH_2_=CH–), 6.27 (1H, CH_2_=CH), 6.40 (1H, CH_2_=CH–), 7.28 (1H, phenyl), 7.49 (1H, phenyl), 7.82 (1H, phenyl), 7.88 (1H, phenyl), 8.03 (2H, –B(OH)_2_, 10.07 (1H, –NH–) (Figure S1A) and ^11^B NMR 28.7(^11^B) (Figure S1B).

The synthesis of boronate-PVA SMPs was achieved by using the oil emulsion method. The fabrication of boronate-PVA SMPs also involved a free radical polymerization of AAPBA and DMAEMA by using APS/TEMED. Boronate forms a complex with PVA by *cis*-diol interactions and the polymeric form of boronate with DMAEMA was formed by free radical polymerization. We anticipated that the binding of boronate with PVA and copolymerization of AAPBA with DMAEMA and HEMA would simultaneously take place. This provides a one-step fabrication method to synthesize boronate-containing particles. The addition of DMAMEA as a tertiary amine protects the boronate group from nucleophilic attack by water molecules. The amino group provided by DMAEMA acts as the lewis base, which donates an electron pair to the vacant *p*-orbital of boron and forms an N→B coordination bond (21). This prevents the surrounding water molecules from forming the tetrahedron boronate configuration.

**Figure 2:**
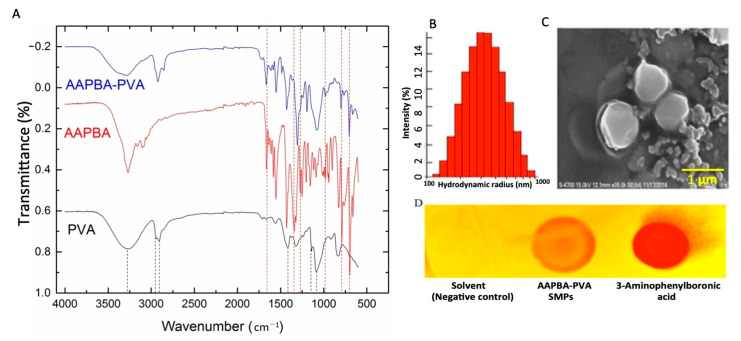
Characterization of the synthesized SMPs. (**A**) Confirmation of the synthesis of AAPBA-polyvinylalcohol (PVA) by Fourier-transform infrared spectroscopy (FTIR). The identified peaks of the chemical groups as follows: 1665 cm^−1^/C=O, 1636 cm^−1^/C=C, Amide II/1555 cm^−1^, Benzene/1429 cm^−1^, Boronate/1347 cm^−1^, OH/3200–3500 cm^−1^. (**B**) Size measurement of AAPBA-PVA SMPs by dynamic light scattering (DLS). (**C**) SEM image of AAPBA-PVA SMPs. (**D**) Functional integrity of boronate confirmed by the development of a red-colored spot in curcumin thin layer chromatography (TLC).

Hence, in the presence of a tertiary amine, the tetrahedral anionic form of boronate becomes active at a neutral pH and governs boronate and *cis*-diol interactions. The size of glucose-responsive SMPs synthesized by this method was 825–875 nm in diameter (Figure 2B,C). The particles had a negative charge, which was −12.2 mV as determined by a zeta sizer. The significant peaks present from both reactants in the FTIR spectra were 3270 cm^−1^/O–H 2905 cm^−1^, and 2940 cm^−1^/C–H alkyl groups, 1413 cm^−1^/CH_2_, 1143 cm^−1^/C–O or C–O–C, 1085 cm^−1^/C–O–C (from PVA), and 1665 cm^−1^/C=O, 1270 cm^−1^/C–H, 1350 cm^−1^/B–OH, 701 cm^−1^/phenyl group (from AAPBA confirms the synthesis of the AAPBA-PVA complex (Figure 2A). Curcumin reacts with several boron-containing species to give a colored complex called a rosocyanine complex. This color is due to complex formation between the boron species and curcumin (24). In our study, curcumin replaced the PVA and interacted with boronate to produce a visible red complex confirmed by TLC (Figure 2D). Hence, the functional integrity of boronate was maintained in the complexed form.

### 3.2. Transfection Studies

#### 3.2.1. Optimization of the Plasmid:Polymer Ratio

**Figure 3:**
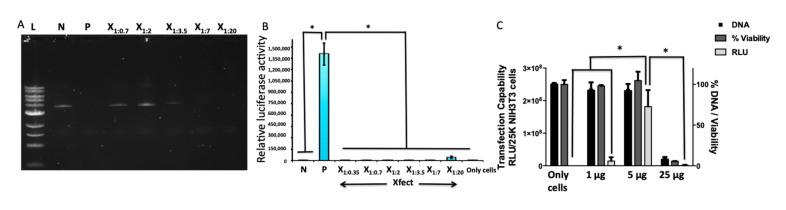
Optimization of transfection efficacy of GLuc polyplex complexation. (**A**) The complexation of plasmid-polymer investigated by agarose gel electrophoresis using different plasmid:polymer ratios; (Lanes 1: Ladder, 2: Naked plasmid, 3: PEI, 4: Xfect 1:0.7, 5: Xfect 1:2, 6: Xfect 1:3.5, 7: Xfect 1:7, 8: Xfect 1:20). (**B**) Analysis of transfection in NIH3T3 cells at different plasmid:polymer (plasmid/polymer) ratios by luciferase assay (Bars 1: Naked plasmid, 2: PEI, 3: Xfect 1:0.35, 4: Xfect 1:0.7, 5: Xfect 1:2, 6: Xfect 1:3.5, 7: Xfect 1:7, 8: Xfect 1:20, 9: Only cells). (**C**) Dose optimization in three different assays, i.e., luciferase assay for cellular transfection, alamarBlue™ assay for metabolic activity, picogreen^®^ assay for DNA quantification, was performed. Results indicate that Xfect™ showed its maximum transfection with minimal toxicity at 5 µg, * represents statistical significance by one-way ANOVA, *n* = 3, SD, and *p* < 0.05.

A different range of plasmid:polymer ratios was used to form polyplexes. At 1:7 and 1:20 plasmid:polymer ratios, the complete complexation of GLuc was observed by agarose gel electrophoresis (Figure 3A). However, transfection of the cells occurred only with the 1:20 ratio (Figure 3B). Therefore, this was the ratio that was used for all further transfection experiments. The commonly used transfection reagent PEI was used as a positive control, and naked plasmid was used as a negative control.

#### 3.2.2. Optimization of Polyplex Dose in NIH3T3 Cells

Three different concentrations of polyplex, i.e., 1, 5 and 25 µg/well, were studied for transfection capability. At 1 µg concentration, the cell viability was good; however, transfection was less (1.4 × 10^7^ relative light units (RLUs)). In total, 25 µg of polyplex was toxic to the cells, and thus minimal transfection was observed at this concentration. A dose of 5 µg of GLuc was optimal for NIH3T3 cells where the highest transfection (1.8 × 10^8^ RLU) was observed (Figure 3C).

### 3.3. Glucose-Responsive Behavior

#### 3.3.1. Hydrodynamic Diameter

**Figure 4:**
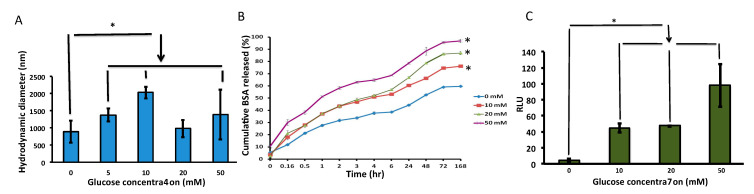
Glucose-responsive behavior of the synthesized SMPs. (**A**) The increase in the hydrodynamic diameter of AAPBA-PVA SMPs corresponded with glucose concentration up to a value of 10mM glucose; after this, the integrity of the SMPs was destabilized because of the high water pressure developed inside them. (**B**) Release of encapsulated bovine serum albumin (BSA) from the synthesized AAPBA-PVA SMPs was depending on the concentration of glucose present at the site. (**C**) The highest relative light unit (RLU) corresponds to highest transfection was observed at the highest glucose concentration—i.e., 50 mM; *n* = 3, SD, *p* < 0.05, and * represents statistical significance by one-Way ANOVA.

To assess the influence of glucose on the AAPBA–PVA complex, the hydrodynamic diameter shift of SMPs was observed at different glucose concentrations ranging from 5 to 50 mM (Figure 4A) DLS. It was found that the increase in the hydrodynamic diameter of SMPs correlated with increasing glucose concentrations. The SMPs swelled from a diameter of 800 nm to a diameter of 2000 nm in the presence of 10 mM glucose concentration at a physiological pH. This increase in diameter is due to a slow deterioration of boronate-PVA. The glucose molecule replaces PVA and allows the inward movement of water into the SMPs, resulting in the degradation of the SMPs. The degradation of SMPs occurred when the glucose concentration increased more than 10 mM. Due to the degradation of the SMPs, the hydrodynamic diameter was reduced at 20 and 50 mM glucose concentrations. This demonstrates that the boronate remains active at a physiological pH which allows the weakening of the boronate–PVA complex in the presence of glucose and, in reverse, allows the formation of the boronate–glucose complex.

#### 3.3.2. Glucose-responsive BSA Release

Different glucose concentrations (0, 10, 50, 100 mM) were studied to validate BSA release from the BSA encapsulated SMPs (Figure 4B). The amount of BSA released was directly proportional to the increase in glucose concentration. The cumulative percentage of BSA released was plotted as a factor of time. It was observed that 75%, 85%, and 95% BSA was released from SMPs at 10, 20 and 50 mM glucose concentrations after 72 h.

#### 3.3.3. Glucose-Responsive Transfection

The GLuc polyplexes were successfully loaded within boronate-PVA SMPs. The release of GLuc polyplexes from the boronate-PVA SMPs correlated with the increasing glucose concentrations. A two-fold increase in transfection corresponding RLUs was observed for 50 mM glucose-containing media compared to 10 or 20 mM glucose-containing media (Figure 4C).

### 3.4. Cytocompatibilty

#### 3.4.1. Metabolic Activity

**Figure 5:**
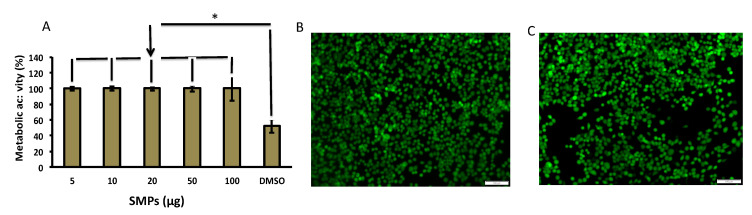
Cytocompatibility of the synthesized SMPs on NIH3T3 cells. (**A**) The cellular metabolic activity in the presence of SMPs was assessed by alamarBlue^TM^ assay, and the assays showed that the metabolic activity was not affected in the presence of SMPs. (**B**) Merged fluorescence image of live cells (green channel) and dead cells (red channel) without SMPs. (**C**) Merged fluorescence image of live cells (green channel) and dead cells (red channel) in the presence of SMPs. Scale bar = 100 μm. *n* = 3, SD, *p* < 0.05, and * represents statistical significance by one-Way ANOVA.

We assessed the cytotoxicity of SMPs in the glucose-containing media on NIH3T3 fibroblast monolayer culture. The cytocompatibility of AAPBA-PVA SMPs was assessed with alamarBlue™ assay. It was found that the metabolic activity of the cells was not affected by the presence of SMPs even after 48 h at the highest concentration used (100 µg/well) (Figure 5A). This demonstrates that the SMPs and their degraded polymer by-products do not cause any short-term cytotoxicity.

#### 3.4.2. Live Dead Assay

The live dead assay confirmed that no cell death was seen in the presence of AAPBA-PVA SMPs after 48 h at 100 µg concentration (Figure 5B,C).

## 4. Conclusions

By utilizing boronate *cis*-diol interactions, a glucose-responsive SMPs system was developed. The stability of the boronate interaction at a physiological pH was achieved by copolymerization of DMAEMA with AAPBA, and *cis*-diol interactions governed the formation of a complex with PVA. The complete fabrication of boronate-containing SMPs was achieved in a single-pot synthesis. The synthesized AAPBA-PVA SMPs exhibited glucose-responsive behavior at higher glucose concentrations in physiological pH conditions. The hydrodynamic diameter of SMPs increased up to 2000 nm in 10 mM glucose, and after that degradation was initiated. This suggests the slow and responsive behavior of SMPs with reversible binding to external glucose molecules. The destabilization of the AAPBA–PVA complex by interacting with glucose allows the release of the encapsulated BSA in a glucose-responsive manner. AAPBA-PVA SMP loaded GLuc genes were delivered to the NIH3T3 cells depending on the glucose concentration present at the delivery site. Finally, the cellular metabolic activity of NIH3T3 cells was not altered in the presence of the SMPs at a concentration of 100 µg/well, as shown in alamarBlue^TM^ assay. Herein, we have successfully presented a fabrication method of glucose-responsive SMPs for gene delivery. However, investigating these SNPs in an in vivo hyperglycemic disease model is necessary to ensure its safety and therapeutic potential.

## Data Availability

Data can be made available on request.

## Supplementary Materials

The following are available online at https://www.mdpi.com/1999-4923/13/1/62/s1, Figure S1: Confirmation of AAPBA synthesis by NMR, FTIR analysis.

## Abbreviations

SMPs—Submicronic particles, AAPBA—Acrylamidophenylboronic acid, PVA—Polyvinyl alcohol, DEMAEMA—*N*,*N*-Dimethylaminoethyl Methacrylate, HEMA—2-Hydroxyethyl methacrylate, APS—Ammonium persulfate, TEMED—*N*,*N*,*N*’,*N*’-Tetramethylethane-1,2-diamine, BSA—Bovine serum albumin, GLuc—Gaussia luciferase, ^1^H NMR—Proton nuclear magnetic resonance, ^11^B NMR—Boron nuclear magnetic resonance, FTIR—Fourier-transform infrared spectroscopy, SEM—Scanning electron microscopy, TLC—Thin layer chromatography, PEI—Polyethylenimine, DLS—Dynamic light scattering, and RLU-Relative light units.
