# Supplementary material for: Glucose-Responsive Gene Delivery at Physiological pH through Tertiary-Amine Stabilized Boronate-PVA Particles Synthesized by One-Pot Reaction
Source: Pharmaceutics. 2021 Jan 6;13(1):62. doi: 10.3390/pharmaceutics13010062 (PMC7825127; doi:10.3390/pharmaceutics13010062)
Supplement: Supplementary file 1 [file pharmaceutics-13-00062-s001.pdf]

pharmaceutics

Supplementary Materials: Glucose-Responsive Gene Delivery at Physiological pH through Tertiary-Amine Stabilized Boronate-PVA Particles Synthesized by One-Pot Reaction

Mangesh Morey, Akshay Srivastava and Abhay Pandit

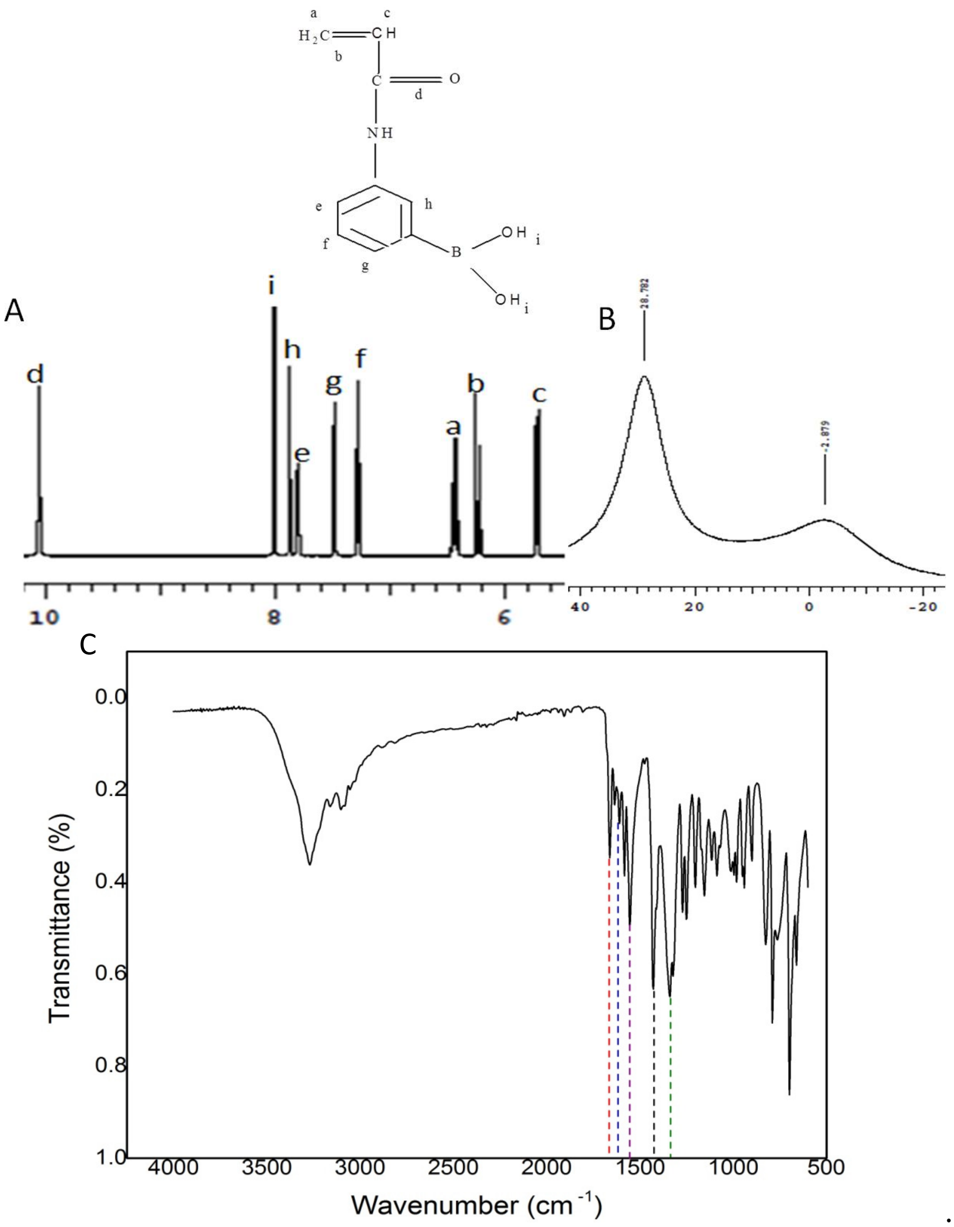

Figure S1. Confirmation of AAPBA synthesis by NMR, FTIR analysis (A) 1H NMR (B) 11B NMR (C) FTIR C=O; δ=1666 cm^{-1}, C=C δ=1636 cm^{-1}, Amide II; δ=1557 cm^{-1}, benzene; δ=1433 cm^{-1}, –B(OH)$_2$; δ=1356 cm^{-1}.
